# Kappa Free Light Chains in Multiple Sclerosis as a Marker of Intrathecal Humoral Response: A Sex-Disaggregated Study

**DOI:** 10.3390/diagnostics14242798

**Published:** 2024-12-12

**Authors:** Raffaella Candeloro, Maila Galloppa, Laura Lombardo, Michele Laudisi, Sara Ghisellini, Giovanna Negri, Caterina Ferri, Carla Marcialis, Tiziana Bellini, Maura Pugliatti, Massimiliano Castellazzi

**Affiliations:** 1 Department of Neurosciences and Rehabilitation, University of Ferrara, 44121 Ferrara, Italy; raffaella.candeloro@unife.it (R.C.); maila.galloppa@edu.unife.it (M.G.); laura.lombardo@edu.unife.it (L.L.); carla.marcialis@edu.unife.it (C.M.); tiziana.bellini@unife.it (T.B.); maura.pugliatti@unife.it (M.P.); 2 Department of Neuroscience, “S. Anna” University Hospital, 44124 Ferrara, Italy; michele.laudisi@ospfe.it (M.L.); caterina.ferri@ospfe.it (C.F.); 3 Clinical Pathology Unit, “S. Anna” University Hospital, 44124 Ferrara, Italy; s.ghisellini@ospfe.it (S.G.); g.negri@ospfe.it (G.N.); 4 University Strategic Center for Studies on Gender Medicine, University of Ferrara, 44121 Ferrara, Italy; 5 Interdepartmental Research Center for the Study of Multiple Sclerosis and Inflammatory and Degenerative Diseases of the Nervous System, University of Ferrara, 44121 Ferrara, Italy

**Keywords:** multiple sclerosis, cerebrospinal fluid, intrathecal synthesis, kappa free light chains, sex differences

## Abstract

Background: Kappa free light chains (KFLCs) are emerging as promising biomarkers for intrathecal B cell activity for diagnosing multiple sclerosis (MS) through cerebrospinal fluid (CSF) analysis. In this study, we evaluated the ability of KFLC formulas to identify the presence of MS and their agreement with the ‘gold standard’ of CSF IgG oligoclonal bands (OCBs). Methods: A total of 233 patients were included in this study: 149, comprising 43 males and 106 females, had MS, and the remainder, 40 males and 44 females, had other neurological diseases (ONDs). We evaluated the potential of KFLCs in terms of sensitivity, specificity, and accordance. All analyses were conducted using a sex-disaggregated approach. Results: KFLCs showed a high sensitivity for both sexes with respect to the diagnosis of MS, with values between 74.42% and 93.03%. The specificity of the various formulas was much lower for females when compared to males, with values between 45.45% and 59.09%, with a significant difference between the two sexes for the K Index > 5.9 (*p* = 0.0451). Cohen’s kappa showed substantial agreement for men and moderate agreement for women between the KFLC indices and OCB. Conclusions: This study highlights the potential of KFLCs as a biomarker for MS but emphasises the need for sex-specific thresholds to improve diagnostic accuracy.

## 1. Introduction

Multiple sclerosis (MS) is a chronic inflammatory immune-mediated disease of the central nervous system (CNS) that mainly affects young adults and poses a risk of physical and cognitive disability [1]. Cerebrospinal fluid (CSF) analysis remains a fundamental aspect of the MS diagnostic process [2], especially after the 2017 revision of the diagnostic criteria. These criteria reaffirm the importance of CSF analysis, along with magnetic resonance imaging (MRI) and a patient’s medical history. In this context, CSF examination plays a crucial role in diagnosing MS when clinical and MRI findings are inconclusive. [3]. CSF is often considered a sort of “liquid biopsy” for brain fluids due to its diagnostic value in clinical neurology [1]. From a laboratory point of view, MS patients are characterised by the presence of an intrathecal humoral immune response, typically represented by the presence of CSF-restricted IgG oligoclonal bands (OCBs) produced by infiltrating B cells [4]. This intrathecal IgG synthesis has been considered the distinctive laboratory feature of patients with MS for years. The “gold standard” for this type of investigation has been identified as the isoelectric focusing (IEF) of paired serum and CSF samples on agarose gel, followed by IgG-specific immunofixation [2]. The assessment of such OCBs remains a highly operator-dependent and time-consuming test.

In recent years, numerous studies have highlighted the potential value of kappa free light chains (KFLC) in CSF as a biomarker for intrathecal B cell activity in MS patients, not least thanks to significant methodological advantages [5]. KFLCs originate from plasma cells, which, in addition to producing whole immunoglobulins, release a proportion of 10–40% of free light chains into the blood, some in dimeric form, some as free lambda light chains, and KFLCs in monomeric form [5,6,7]. The KFLCs detectable in the CSF, as with CSF IgG, can have a double origin: they can be plasma-derived, after simply having crossed the blood–CSF barrier (BCSFB) according to a concentration gradient, or intrathecal, i.e., produced by plasma cell clones active in the CNS [8]. In recent years, different indices have been proposed to quantify the intrathecal production of KFLCs. Some of these mathematical formulas are linear, such as the K index, and others are non-linear, like Reiber’s formula [9]. Different thresholds have been indicated for K Index; however, an agreement on their application is still lacking.

An important emerging consideration is the sex-related difference in CSF analyses [10]. Males generally have higher CSF density and protein content compared to females [11]. Additionally, males exhibited higher BCSFB permeability, evaluated through the quotient of albumin (QAlb), across various conditions, including SM [12], amyotrophic lateral sclerosis [13], and psychiatric disorders [14], and in healthy individuals [15]. Of particular interest are the potential implications of these sex-related differences for the formulas proposed for the evaluation of intrathecal KFLC synthesis since the QAlb is indeed included in all the formulas proposed to date.

This study aims to assess the accuracy of KFLCs in diagnosing MS and compare its results to the ‘gold standard’ IgG oligoclonal bands, considering potential sex differences in blood–cerebrospinal fluid barrier permeability.

## 2. Materials and Methods

### 2.1. Study Design

This retrospective monocentric study included patients who had undergone lumbar puncture for diagnostic purposes at the S. Anna University Hospital in Ferrara between 2018 and 2024. This study was approved by the Medical Ethics Committee for Research “Comitato Etico di Area Vasta Emilia Centro della Regione Emilia-Romagna” (protocol number 770/2018/Oss/AOUFe). This study did not involve any interference with diagnostic or therapeutic procedures of good clinical practice, nor did it involve any additional procedures. Samples were anonymised before examination. Written informed consent was obtained from each patient upon admission.

### 2.2. Study Population

This study included 233 neurological patients: 83 males and 150 females. The inclusion criteria for the MS group required a definite diagnosis of MS, while the control group consisted of consecutive patients with confirmed clinical diagnoses of various neurological disorders. Accordingly, all data collected, analyses performed, and anamnestic and clinical information related to the time of lumbar puncture. Of the total 233 patients, 149 had MS, comprising 43 males and 106 females, and the rest consisted of a cohort of real-world patients with other neurological diseases (ONDs), on whom data were collected between June 2022 and June 2024, comprising 11 patients with clinically isolated syndromes (CIS), comprising 5 males and 6 females; 1 patient with radiologically isolated syndrome (RIS), comprising 1 female; 27 inflammatory neurological disease controls (INDCs), comprising 11 males and 16 females; 14 peripheral inflammatory neurological disease controls (PINDCs), comprising 9 males and 5 females; and 31 patients with non-inflammatory neurological diseases (NINDCs), comprising 15 males and 16 females [16].

Exclusion criteria were CSF white blood cell levels > 10/µL, the presence of CSF discolorations, and having had repeated samples of CSF extracted [12].

### 2.3. Sampling

Paired blood and CSF samples were taken from all patients and shipped to our laboratory within two hours [1]. Samples were centrifuged at 3000× *g* for 10 min at 20 °C to separate the liquid component from the cellular residue. The supernatants obtained were then aliquoted into 0.5 mL volumes and, if not analysed immediately, stored at −80 °C until analysis. All samples were collected, stored, and analysed under the same conditions.

### 2.4. Quantitative Analyses

Serum and CSF concentrations of albumin, IgG, and KFLCs were measured in all samples via turbidimetry using Optilite^®^ (The Binding Site, Birmingham, UK). The following kits were used for the respective assays: (i) for the measurement of albumin levels, the Optilite^®^ Low-Level Albumin Kit was used, with a detection rate in CSF of 11–16,625 mg/L and in serum of 2200–66,500 mg/L (The Binding Site, Birmingham, UK); (ii) for the measurement of IgG levels, the Optilite^®^ Low-Level IgG Kit was used, with a detection rate in CSF of 7.5–1350 mg/L and in serum of 1500–27,000 mg/L (The Binding Site, Birmingham, UK); and (iii) to quantify KFLCs, the Optilite^®^ Kappa Free Light Chains Kit, with a detection rate of 0.33–127,000 mg/L in CSF and serum (The Binding Site, Birmingham, UK), was used.

The QAlb was calculated according to the following formula: QAlb = [albumin]_CSF_ ÷ [albumin]_serum_ [2]. Two different age-dependent formulas were utilised to calculate the upper reference limit (URL) of QAlb: URL = age/15 + 4 [17] and URL = age/25 + 8 [18].

Intrathecal IgG synthesis was determined by calculating the IgG Index using the following formula: IgG Index = QIgG/QAlb [2], where QIgG = [IgG]_CSF_ ÷ [IgG]_serum_. Positive IgG Index values were considered if they were greater than 0.7.

The K Index is determined by the following formula [6]: K Index = ([KFLC]_LCS_/[KFLC]_serum_) ÷ QAlb.

Different thresholds were used according to the literature: 5.9 [19]; 6.1 [7]; 6.61 [6]; and 10.61 [20]. Values above these thresholds were considered suggestive of intrathecal KFLC synthesis.

A non-linear formula was also used for the quantification of intrathecal KFLC levels: Kloc = [Qkappa(total) − Qkappa(lim)] × [KFLC]_serum_ [21]. Here, Qkappa(lim) is calculated as follows: Qkappa(lim) = 3.27 [QAlb^2^ + 33]^0.5^ − 8.2(×10^−3^). The threshold of normality of Kloc values was 0. Higher values were considered suggestive of intrathecal KFLC synthesis.

### 2.5. Qualitative Analyses

At the time of lumbar puncture and for the purpose of diagnosis, in all patients, IgG oligoclonal bands were determined in paired CSF and serum samples using the gold standard for IEF, followed by IgG-specific immunoblotting using the Helena Biosciences IgG IEF kit (Helena Biosciences Europe, Gateshead, UK) [3]. SAS-3 and the SAS IgG IEF kit (Helena Biosciences, Gateshead, UK) were used. The immunoblotting patterns were first independently assessed by two experienced operators, both of whom were blinded, regarding clinical information, and finally the presence/absence of IgG oligoclonal bands (OCBs) was established through interobserver agreement. The following CSF patterns were considered [2]: pattern 1 = absence of IgG OCBs; pattern 2 = 2 or more CSF-restricted IgG OCBs; pattern 3 = 2 or more CSF-restricted IgG OCBs with additional identical IgG OCBs in CSF and serum; pattern 4 = 2 or more identical IgG OCBs in CSF and serum; and pattern 5 = some identical IgG OCBs in CSF and serum in a restricted pH range. Only patterns 2 and 3 indicate an intrathecal IgG synthesis [2].

### 2.6. Statistical Analysis

All data were preliminarily checked for normality using the Kolmogorov–Smirnov test. Continuous variables showing a normal distribution are presented as means and standard deviations (SDs), and the Student’s t-test was used to compare groups, while variables that did not show a normal distribution were presented as medians and interquartile ranges (IQRs), and the Mann–Whitney U test was used for comparison between groups. Categorical variables were reported as counts (percentages), and Fischer’s exact test was used for comparison. The sensitivity and specificity of each parameter in identifying the presence of intrathecal IgG synthesis or making a diagnosis of MS were calculated using 2 × 2 contingency tables, according to a method reported in a previous study [22]. The agreement between quantitative and qualitative methods for identifying an intrathecal humoral response and between different methods for identifying the diagnosis of MS was calculated using Cohen’s kappa coefficient [23] with a free tool: https://www.graphpad.com/quickcalcs/kappa1/ (accessed on 10 December 2024).

Kappa values were interpreted as follows: 0.00–0.20, slight agreement; 0.21–0.40, fair agreement; 0.41–0.60, moderate agreement; 0.61–0.80, substantial agreement; and 0.81–1.00, almost perfect agreement. Two-tailed *p*-values of less than 0.05 were considered statistically significant. Prism 10 software for MacOS (GraphPad Software, La Jolla, CA, USA) was used for the statistical analyses.

## 3. Results

### 3.1. Cerebrospinal Fluid, Serum, and Quantitative Indices of Albumin, IgG, and KFLCs in Sex-Disaggregated Multiple Sclerosis Patients and Controls

When the spinal taps were conducted, there were no differences in age between males and females in the MS (42.70 vs. 40.01 years, *p* = 0.2575) and OND (58.20 vs. 53.59 years, *p* = 0.1795) subgroups.

As reported in Table 1, in the MS subgroup, male patients had significantly higher albumin concentrations in their CSF (252.2 vs. 189.0 mg/L, *p* = 0.0005) and serum (48.40 vs. 44.30 g/L, *p* = 0.0004) compared to females. QAlb values were also higher in males than in females (5.5 vs. 4.535, *p* = 0.0130). These differences were not confirmed in the OND group, where only a higher level of serum KFLCs in women than in men was found (19.39 vs. 14.88 *p* = 0.0150). No significant differences were observed in the two subgroups, for the other parameters of this study.

### 3.2. Positivity of Quantitative Indices of Albumin, IgG, and KFLCs in Sex-Disaggregated Multiple Sclerosis Patients and Controls

As reported in Table 2, positivity for QAlb thresholds was present more frequently in male MS patients than in female MS patients: ‘age/15 + 4’ formula (34.9% vs. 14.2%, *p* = 0.0065) and ‘age/25 + 8’ formula (14% vs. 3.8%, *p* = 0.0342). No differences between sexes in the MS subgroup were found for the IgG Index, the various thresholds for KFLCs, and the presence of CSF-restricted OCBs.

In the OND subgroup, there was only different positivity for the K-index > 5.9, where females showed a higher percentage than males (50% vs. 27.5% *p* = 0.0451). No further statistically significant difference was found in the OND subgroup.

### 3.3. The Ability of KFLC Indices and IgG Oligoclonal Bands to Identify the Diagnosis of Multiple Sclerosis

**Figure 1:**
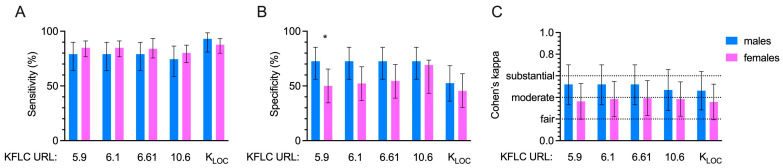
The ability of different indices of free kappa light chains (KFLC) to identify the diagnosis of multiple sclerosis was assessed. Sensitivity (**A**), specificity (**B**), and agreement (**C**) were evaluated for each parameter in the sex-disaggregated population. Abbreviations: URL—Uniform Resource Locator; fair agreement—range from 0.21 to 0.40; moderate agreement—range from 0.41 to 0.60; substantial agreement—range from 0.61 to 0.80. Fisher’s exact test was used to compared sensibility and specificity between males and females. *: *p* = 0.0451.

The performances of the KFLC formulas and OCB with respect to the final diagnosis of MS were evaluated in regard to patients disaggregated by sex. In Figure 1 are reported data on the sensitivity, specificity, and Cohen’s coefficient for each parameter considered.

In particular, K indexes and K_LOC_ sensitivities ranged between 74.42% (K Index > 10.61) and 93.03% (K_LOC_) for males and between 80.19% (K Index > 10.61) and 87.84% (K_LOC_) for females. No differences were found between sexes for sensitivity (Figure 1, panel A).

Specificities had values ranging between 52.5% (K_LOC_) and 72.5% (all K Indexes) for males and between 45.45% (K_LOC_) and 59.09% (K Index > 10.61) for females, with a significant difference between the two sexes for the K Index > 5.9 (*p* = 0.0451) (Figure 1, panel B).

The agreement between methods in identifying the diagnosis of MS was checked using Cohen’s Kappa values. Accordingly, all the parameters showed a ‘moderate agreement’ for males, with a range of 0.462 (K_LOC_) to 0.52 (K indexes >5.9, >6.1, >6.61), while for females, all these variables showed ‘fair agreement’, ranging from 0.358 (K_LOC_) to 0.393 (K index > 6.61) (Figure 1, panel C).

### 3.4. Cerebrospinal Fluid, Serum, and Quantitative Indices of Albumin, IgG, and KFLCs for Sex-Disaggregated Patients Grouped by OCB Positivity

We further subdivided the population’s laboratory data, according to the presence or absence of CSF-restricted IgG OCB, in the OCB+ (37 males and 94 females) and OCB- (46 males and 56 females) groups (Table 3).

In the OCB+ subgroup, male subjects had significantly higher CSF and serum albumin concentrations than females, i.e., 245.8 vs. 229.5 mg/L (*p* = 0.0317) and 48.4 vs. 44.3 g/L (*p* = 0.0035) respectively. In the OCB- subgroup, males had higher CSF albumin and IgG concentrations than females, 287.2 vs. 245.0 mg/L (*p* = 0.0008) and 36.55 vs. 27.35 mg/L (*p* = 0.0096) respectively. Moreover, males showed higher KFLC concentrations in serum than females: 18.58 vs. 14.01 mg/L (*p* = 0.0052). Finally, men had greater QAlb values than females: 6.595 vs. 5.38 (*p* = 0.0019).

No further statistically significant differences were found between males and females in the OCB+ and OCB- subgroups.

### 3.5. Positivity of Quantitative Indices of Albumin, IgG, and KFLC for Sex-Disaggregated Patients Grouped by OCB Positivity

As reported in Table 4, no significant differences were found between males and females in terms of age-adjusted QAlb thresholds, IgG index, or different KFLC indices in the OCB+ and OCB- subgroups.

### 3.6. Inter-Rater Reliability of KFLC Indices and IgG Oligoclonal Bands

The performances of the KFLC indices in identifying an intrathecal inflammatory condition were tested with respect to the ‘gold standard’ for OCBs for patients disaggregated by sex. Sensitivities, specificities, and Cohen’s coefficients were calculated for each KFLC formula.

**Figure 2:**
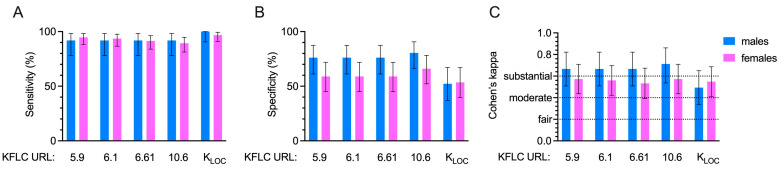
The ability of different indices of free kappa light chains (KFLC) to identify the presence of intrathecal immunoglobulin synthesis was assessed using cerebrospinal-fluid-restricted oligoclonal IgG bands as the reference “gold standard”. Sensitivity (**A**), specificity (**B**), and agreement (**C**) were evaluated for each parameter in the sex-disaggregated population. Abbreviations: URL: Uniform Resource Locator; fair agreement: range from 0.21 to 0.40; moderate agreement: range from 0.41 to 0.60; substantial agreement: range from 0.61 to 0.80. Fisher’s exact test was used to compared sensibility and specificity between males and females: all *p* > 0.05.

As reported in Figure 2, sensitivities ranged between 91.89% (all K Indexes) and 100% (K_LOC_) for males and between 89.36% (K Index > 10.61) and 96.81% (K_LOC_) for females. No differences were found between sexes regarding sensitivity (Figure 2, panel A).

Specificities showed values ranging between 52.17% (K_LOC_) and 80.43% (K Index > 10.61) among males and from 53.57% (K_LOC_) to 66.07% (K Index > 10.61) among females, with no significant differences between the two sexes (Figure 2, panel B).

The agreement between the KFLC formulas and OCB was checked using Cohen’s Kappa. Accordingly, as expected for the K_LOC_, which showed ‘moderate agreement’ (Cohen’s Kappa = 0.493), all parameters showed ‘substantial agreement’ in regard to males, with a range of 0.666 (K Index > 5.9, >6.1, >6.61) to 0.712 (K index > 10.61), while for females, all these variables showed ‘moderate agreement’, with a range of 0.533 (K Index > 6.61) to 0.573 (K index > 5.9, >10.61) (Figure 2, panel C).

### 3.7. No-Multiple-Sclerosis and CSF-Restricted OCB-Negative Subjects Positive for KFLCs

We analysed the OND patients who tested positive for at least one of the KFLC indices to identify potential common profiles among these patients.

Among the 19 male OND patients who yield positive results for at least one KFLC index, 4 had CIS, 8 were INDCs, 4 were NINDCs, and 3 were PINDCs. In the OND female subgroup, 24 had positive results for at least one KFLC index, 4 had CIS, 14 were INDCs, 3 were NINDCs, and 3 were PINDCs.

The same screening was performed for the OCB- patients. Within the male subgroup, 12 yielded positive results for at least one KFLC formula, and 7 had pattern 1, with an absence of IgG OCBs (2 with CIS, 1 INDCS, 3 NINDCs, and 1 PINDC), while 5 patients had pattern 4, and there were 2 or more identical IgG OCBs in CSF and serum (2 PINDCs, 2 INDCs, and 1 NINDC). In the OCB- female subgroup, eight had at least one KFLC-positive index; of these, four had pattern 1 (1 with CIS, 2 INDCs, and 1 PINDC), and four showed pattern 4 (2 INDCs and 2 PINDCs).

## 4. Discussion

Our study demonstrates sex-based differences in KFLC diagnostic performance, highlighting the need for a sex-disaggregated approach to MS diagnosis. In our population of MS patients and real-world OND controls, we observed that the KFLC indices and the K_LOC_ formula had high sensitivities for the diagnosis of MS in both sexes, with values exceeding 90% for men when using the K_LOC_ formula. However, the specificity of these indices was lower for women than for men, with K_LOC_ having the lowest specificity, with values below 50%. This suggests that although KFLC biomarkers are sensitive in detecting MS-associated intrathecal inflammation, they may lead to false positives, especially for women and if used alone. To date, there is no consensus on the optimal threshold, and our study does not aim to identify a definitive value. However, based on our analyses, a threshold of 10.61 appears to offer the best balance between sensitivity and specificity, although the corresponding performance may vary between sexes.

A possible explanation for the different performances of these indices with respect to the two sexes could be the inclusion of the QAlb, commonly used to assess barrier dysfunction, in linear and non-linear formulas for the detection of intrathecal KFLC synthesis. It is known that the QAlb tends to increase with age and shows significant variations between males and females. Indeed, the literature reveals sexual dimorphism for this parameter, with a consequent higher probability of barrier dysfunction in males than in females [12]. This difference in QAlb values could explain the results obtained, in which the KFLC formulas yielded different results for the two sexes. This difference in the performance of the KFLC formulas is reflected in their differing agreement for the two sexes regarding the ability to diagnose MS and agree with the presence of CSF OCB.

The observed sex-specific differences in KFLC specificity suggest that sex-adjusted thresholds for KFLCs could improve diagnostic accuracy in MS, particularly for women, and thus minimise false-positive results for other neurological conditions.

The ability of different indices of KFLC to identify the presence of intrathecal immunoglobulin synthesis was also assessed while blind to diagnosis but while using IgG OCB as the ‘gold standard’ of reference. Also, in this case, KFLC formulas showed lower agreement for females than for males, where specificity had consistently lower values than for men, with the sole exception of the K_LOC_ formula, which had similar specificity values in both sexes.

Among the OCB- patients, many yielded positive results for at least one KFLC index, suggesting that these biomarkers may reflect general inflammatory states and not be exclusively related to MS. Indeed, it has been hypothesised that the presence of KFLCs may be linked to other CNS pathologies characterised by an inflammatory state. A previous study demonstrated that CNS infections were the most frequent pathologies in OCB- patients showing a KFLC positive profile [19]. It is important to emphasise that KFLCs are not specific for IgG, as they also correspond to IgM, IgA, IgD, and IgE [19]. In this context, KFLC positivity may precede a formal MS diagnosis, potentially identifying patients in the acute inflammatory phase or those with clinically isolated syndrome (CIS) [19,24].

In our study, it is interesting to note that 9 out of the 13 patients with OCB pattern 4 were positive with respect to at least one KFLC formula. By definition, pattern 4 is characterised by identical oligoclonal bands in CSF and serum samples, indicating a systemic, and not intrathecal, immune response [2]. This profile was associated with an abnormal BCSFB in five out of nine patients, comprising three males and two females, of whom two were PINDCs, two were INDCs, and one was an NINDC.

Considering the limitations regarding the specificity of KFLCs, it is necessary to interpret the results with caution or in tandem with a critical assessment by a clinician. Moreover, the combined use of KFLCs and OCBs could reduce the possibility of obtaining false-positives results, as suggested in recent articles [24,25]. Our data seem to suggest caution is necessary in the application of the K-index and its thresholds, especially for patients with suspected infectious or inflammatory CNS diseases. The intrathecal synthesis of KFLCs, as assessed by means of the K Index, offers numerous advantages over the current ‘gold standard,’ CSF IgG [7]. In particular, analysis via turbidimetry is a faster method than the laborious IEF process used to detect OCBs. The latter is a manual, time-consuming, and operator-dependent technique. The KFLC measurement process is fully automated, reducing operator intervention and minimising the risk of errors in the interpretation of results, which, for OCBs, is carried out through inter-observer agreement.

Another fundamental aspect explored in our study is the comparison of the different diagnostic thresholds for the K Index that have been proposed in the literature, without any particular indication as to which is the most reliable [19,24]. In fact, based on previous works, no unambiguously applied cut-off has yet emerged, which represents a challenge in the application of this parameter in diagnostic routines.

Our work highlights how the KLOC [26] is the threshold with the highest sensitivity among the other indices, albeit at the expense of lower specificity.

While OCB analysis is a more time-consuming and manual process, it remains crucial for MS diagnoses owing to its high specificity. For decades, OCBs have been considered the ‘gold standard’ for detecting intrathecal IgG synthesis, the hallmark of chronic CNS inflammation, and it is probable that their use in combination with KFLCs is important for developing a precision laboratory diagnostic of MS [24,25].

The present study has some limitations. The primary limitations are its sample size and monocentric nature. The MS patient cohort consisted of individuals hospitalised within the past decade, while the neurological control group comprised real-world patients recruited between June 2022 and June 2024. Furthermore, in order to go into detail on the specificity and sensitivity aspects, it would have been helpful to include a healthy population and a population of patients with CIS, respectively. Unfortunately, lumbar punctures are currently primarily performed for diagnostic purposes. The minimally invasive nature of the procedure used limited our ability to obtain cerebrospinal fluid from healthy individuals, preventing the establishment of a healthy control group.

## 5. Conclusions

Our study confirms that the KFLC formulas offer high sensitivity for the diagnostic framing of patients with suspected MS. However, these indices have low specificity, especially for females, resulting in an increased incidence of false-positive results. Although our findings indicate the potential of KFLCs to replace OCBs, future studies should focus on developing sex-specific thresholds and investigating the lower specificity for females, a population with greater MS susceptibility. This could allow us to optimise and better clarify the role of KFLCs in the diagnosis of MS and in the identification of intrathecal humoral responses.

## Figures and Tables

**Table 1:** Cerebrospinal fluid (CSF), serum, and quantitative indices of albumin (QAlb), IgG (IgG Index), and kappa free light chains (KFLC) (K Index) in sex-disaggregated multiple sclerosis (MS) patients and patients with other neurological diseases (ONDs).

	Males	Females	*p*
MS patients: *n*	43	106	
CSF albumin (mg/L): median (IQR)	252.2 (191.1–341.1)	189.0 (142.5–254.5)	**0.0005**
Serum albumin (g/L): median (IQR)	48.40 (45.65–51.49)	43.7 (40.78–47.93)	**0.0004**
QAlb × 10^−3^: median (IQR)	5.5 (3.8–7.5)	4.535 (3.403–5.640)	**0.0130**
CSF IgG (mg/L): median (IQR)	41.70 (26.40–60.40)	34.75 (25.25–52.68)	0.2899
Serum IgG (g/L): median (IQR)	10.09 (8.904–12.14)	10.45 (9.1–12.43)	0.2338
IgG Index: median (IQR)	0.6780 (0.551–0.858)	0.715 (0.5775–0.9175)	0.4329
CSF KFLC (mg/L): median (IQR)	2.050 (0.77–3.830)	1.715 (0.7775–5.295)	0.9127
Serum KFLC (mg/L): median (IQR)	12.74 (10.02–16.12)	14.17 (10.31–17.17)	0.3435
K Index: median (IQR)	24.80 (8.0–84.20)	27.75 (13.28–80.08)	0.8501
OND patients: *n*	40	44	
CSF albumin (mg/L): median (IQR)	291.0 (247.3–374.5)	274.0 (230.5–329.5)	0.2892
Serum albumin (g/L): median (IQR)	45.09 (37.77–48.48)	42.40 (37.70–47.73)	0.4418
QAlb × 10^−3^: median (IQR)	6.62 (5.268–9.460)	6.660 (5.220–7.863)	0.3523
CSF IgG (mg/L): median (IQR)	37.5 (29.45–68.35)	41.35 (25.53–68.43)	0.7232
Serum IgG (g/L): median (IQR)	11.07 (9.342–12.97)	10.75 (9.20–12.75)	0.7199
IgG Index: median (IQR)	0.5500 (0.4425–0.6335)	0.565 (0.472–0.680)	0.3251
CSF KFLC (mg/L): median (IQR)	0.505 (0.2825–1.205)	0.540 (0.000–2.158)	0.7913
Serum KFLC (mg/L): median (IQR)	19.39 (13.89–34.67)	14.88 (11.34–21.23)	**0.0150**
K Index: median (IQR)	1.350 (0.8250–13.13)	5.40 (0.000–14.55)	0.7292

Abbreviations: IQR, interquartile range. Mann–Whitney test was used to compare sexes. Bold *p*-values indicate statistical significance.

**Table 2:** Positivity of quantitative indices of albumin (QAlb), IgG (IgG Index), kappa free light chains (KFLCs) (K index and QKFLC_LOC_), and presence of cerebrospinal-fluid-restricted IgG oligoclonal bands (OCBs) in sex-disaggregated multiple sclerosis (MS) patients and patients with other neurological diseases (ONDs).

	Males	Females	*p*
MS patients: *n*	43	106	
QAlb > age/15 + 4: n (%)	15 (34.9%)	15 (14.2%)	**0.0065**
QAlb > age/25 + 8: n (%)	6 (14%)	4 (3.8%)	**0.0342**
IgG Index > 0.7: n (%)	19 (44.2%)	56 (52.8%)	0.3700
K Index > 5.9: n (%)	34 (79.1%)	90 (84.9%)	0.4684
K Index > 6.1: n (%)	34 (79.1%)	90 (84.9%)	0.4684
K Index > 6.61: n (%)	34 (79.1%)	89 (83.9%)	0.4822
K Index > 10.61: n (%)	32 (74.41%)	85 (80.18%)	0.5098
QKFLCLOC > 0: n (%)	40 (93.0%)	93 (87.73%)	0.5595
Intrathecal IgG OCB: n (%):	30 (69.8%)	78 (73.6%)	0.6873
OND patients: *n*	40	44	
QAlb > age/15 + 4: n (%)	13 (32.5%)	22 (50%)	0.1245
QAlb > age/25 + 8: n (%)	8 (20%)	8 (18.18%)	>0.9999
IgG Index > 0.7: n (%)	7 (17.50%)	10 (22.73%)	0.5969
K Index > 5.9: n (%)	11 (27.5%)	22 (50%)	**0.0451**
K Index > 6.1: n (%)	11 (27.5%)	21 (47.7%)	0.0732
K Index > 6.61: n (%)	11 (27.5%)	20 (45.45%)	0.1145
K Index > 10.61: n (%)	11 (27.5%)	18 (40.9%)	0.2524
QKFLCLOC > 0: n (%)	19 (47.5%)	24 (54.55%)	0.6624
Intrathecal IgG OCB: n (%):	7 (17.50%)	16 (36.36%)	0.0853

Fisher’s exact test was used to compare sexes. Bold *p*-values indicate statistical significance.

**Table 3:** Cerebrospinal fluid (CSF), serum, and quantitative indices of albumin (QAlb), IgG (IgG Index), and kappa free light chains (KFLC) (K Index) in the sex-disaggregated population grouped by the presence (+) or the absence (−) of cerebrospinal-fluid-restricted oligoclonal IgG bands (OCBs).

	Males	Females	*p*
OCB+: *n*	37	94	
CSF albumin (mg/L): median (IQR)	245.8 (193.3–328.7)	229.5 (153.3–282.5)	**0.0317**
Serum albumin (g/L): median (IQR)	48.4 (44.25–50.55)	44.3 (40.1–48.13)	**0.0035**
QAlb × 10^−3^: median (IQR)	5.21 (3.925–7.225)	4.915 (3.588–6.128)	0.1726
CSF IgG (mg/L): median (IQR)	43.6 (29.95–61.75)	42.1 (28.6–59.48)	0.8397
Serum IgG (g/L): median (IQR)	9.905 (8.919–11.79)	10.55 (9.4–12.9)	0.0716
IgG Index: median (IQR)	0.734 (0.61–1.025)	0.75 (0.61–1.033)	0.6941
CSF KFLC (mg/L): median (IQR)	2.51 (1.545–4.765)	2.325 (1.085–7.333)	0.8417
Serum KFLC (mg/L): median (IQR)	13.41 (10.25–15.5)	14.68 (10.64–17.43)	0.1390
K Index: median (IQR)	33.2 (22.35–85.15)	40.05 (15.78–92.03)	0.9179
OCB−: *n*	46	56	
CSF albumin (mg/L): median (IQR)	287.2 (249.2–391.0)	245.0 (162.8–292.8)	**0.0008**
Serum albumin (g/L): median (IQR)	45.93 (38.67–49.18)	43.5 (40.23–46.65)	0.4262
QAlb × 10^−3^: median (IQR)	6.595 (5.228–10.07)	5.38 (3.638–7.25)	**0.0019**
CSF IgG (mg/L): median (IQR)	36.55 (26.58–67.45)	27.35 (19.05–47.78)	**0.0096**
Serum IgG (g/L): median (IQR)	10.87 (9.287–13.05)	10.45 (8.85–12.28)	0.2619
IgG Index: median (IQR)	0.52 (0.44–0.606)	0.52 (0.4625–0.6075)	0.5633
CSF KFLC (mg/L): median (IQR)	0.465 (0.0–0.765)	0.37 (0.0–0.86)	0.4008
Serum KFLC (mg/L): median (IQR)	18.58 (12.39–29.34)	14.01 (10.56–19.34)	**0.0052**
K Index: median (IQR)	1.35 (0.0–6.2)	1.45 (0.0–14.38)	0.7655

Abbreviations: IQR, interquartile range. Mann–Whitney test was used to compare sexes. Bold *p*-values indicate statistical significance.

**Table 4:** Positivity of quantitative indices of albumin (QAlb), IgG (IgG Index), and kappa free light chains (KFLCs) (K indexes and QKFLC_LOC_) in sex-disaggregated patients grouped according to the presence (OCB+) or absence (OCB−) of cerebrospinal-fluid-restricted IgG oligoclonal bands (OCBs).

	Males	Females	*p*
**OCB+: *n***	**37**	**94**	
QAlb > age/15 + 4: n (%)	12 (32.4)	17 (18.1)	0.1011
QAlb > age/25 + 8: n (%)	3 (8.1)	6 (6.4)	0.7116
IgG Index > 0.7: n (%)	21 (56.8)	57 (60.6)	0.6970
K Index > 5.9: n (%)	34 (91.9)	89 (94.7)	0.6865
K Index > 6.1: n (%)	34 (91.9)	88 (93.6)	0.7116
K Index > 6.61: n (%)	34 (91.9)	86 (91.5)	>0.9999
K Index > 10.61: n (%)	34 (91.9)	84 (89.4)	>0.9999
QKFLCLOC > 0: n (%)	37 (100)	91 (96.8)	0.5583
**OCB−: *n***	**46**	**56**	
QAlb > age/15 + 4: n (%)	16 (34.8)	13 (23.2)	0.2702
QAlb > age/25 + 8: n (%)	11 (23.9)	6 (10.7)	0.1086
IgG Index > 0.7: n (%)	5 (10.9)	6 (10.7)	>0.9999
K Index > 5.9: n (%)	11 (23.9)	23 (41.1)	0.0914
K Index > 6.1: n (%)	11 (23.9)	23 (41.1)	0.0914
K Index > 6.61: n (%)	11 (23.9)	23 (41.1)	0.0914
K Index > 10.61: n (%)	9 (19.6)	19 (33.9)	0.1230
QKFLCLOC > 0: n (%)	22 (47.8)	26 (46.4)	>0.9999

Fisher’s exact test was used to compare sexes. Bold *p*-values indicate statistical significance.

## Data Availability

The datasets used and analysed during the current study are available from the corresponding author upon reasonable request.
